# A Simple Cardiovascular Model for the Study of Hemorrhagic Shock

**DOI:** 10.1155/2020/7936895

**Published:** 2020-12-24

**Authors:** Luciano Curcio, Laura D'Orsi, Fabio Cibella, Linn Wagnert-Avraham, Dean Nachman, Andrea De Gaetano

**Affiliations:** ^1^National Research Council of Italy, Institute for Biomedical Research and Innovation, Via Ugo La Malfa, 153, 90146 Palermo, Italy; ^2^National Research Council of Italy, Institute for Systems Analysis and Computer Science “A. Ruberti”, BioMatLab (Biomathematics Laboratory), UCSC Largo A. Gemelli 8, 00168 Rome, Italy; ^3^Institute for Research in Military Medicine (IRMM), Faculty of Medicine, The Hebrew University of Jerusalem and the Israel Defense Forces Medical Corps, Israel; ^4^Department of Internal Medicine, Hadassah University Hospital, Jerusalem, Israel

## Abstract

Hemorrhagic shock is the number one cause of death on the battlefield and in civilian trauma as well. Mathematical modeling has been applied in this context for decades; however, the formulation of a satisfactory model that is both practical and effective has yet to be achieved. This paper introduces an upgraded version of the 2007 Zenker model for hemorrhagic shock termed the ZenCur model that allows for a better description of the time course of relevant observations. Our study provides a simple but realistic mathematical description of cardiovascular dynamics that may be useful in the assessment and prognosis of hemorrhagic shock. This model is capable of replicating the changes in mean arterial pressure, heart rate, and cardiac output after the onset of bleeding (as observed in four experimental laboratory animals) and achieves a reasonable compromise between an overly detailed depiction of relevant mechanisms, on the one hand, and model simplicity, on the other. The former would require considerable simulations and entail burdensome interpretations. From a clinical standpoint, the goals of the new model are to predict survival and optimize the timing of therapy, in both civilian and military scenarios.

## 1. Introduction

Hemorrhagic shock is the number one cause of death both on the battlefield and in civilian trauma. During the Vietnam War, it was found that approximately 50% of deaths were caused by exsanguination [1]. In civilian life, trauma and bleeding cause more deaths in young adults than all other causes combined [2]. Fatal hemorrhage represents a substantial global problem, with more than 60,000 deaths annually in the United States alone and an estimated 1.9 million deaths worldwide, 1.5 million of which are due to trauma [2, 3].

Emergency treatment of severe hemorrhage is a common medical emergency, not infrequently leading to hemorrhagic shock. The current mainstay of hemorrhagic shock treatment consists in whole blood infusions, often large volumes over a short time, termed massive transfusion (MT). Furthermore, survivors of severe hemorrhage have poorer prognoses in terms of functional outcomes and significantly increased mortality [4, 5]. One approach to improving outcomes could focus on shortening the time intervals before diagnosing hemorrhagic shock.

In order to offer healthcare providers with critical decision-making support tools, we needed to create a reliable mathematical model. Ease of use would have to be a key feature allowing practitioners to readily evaluate the individual patient and optimize available therapeutic strategies so as to limit the need for massive transfusions.

Many studies have focused on developing experimental models of hemorrhagic shock to investigate its pathophysiology and to test therapeutic options [6, 7]. However, translating experimental results into clinical applications has remained an ongoing challenge [8].

A variety of hemorrhagic shock models have been designed contemplating controlled or uncontrolled bleeding scenarios or combining hemorrhagic shock and polytrauma [9, 10]. The effectiveness of such mathematical models depends on the reliability in a patient-centered perspective. In order to be predictive, computational simulations require corroborating clinical data. To this end, key model parameters and initial and boundary conditions are estimated minimizing errors between model outputs and empirical measurements (i.e., by solving the so-called “inverse problem” [11]).

Our model is essentially based on the one created by Zenker et al. [12] (albeit revised and upgraded). Zenker's model [12], among all currently available models, appears to offer the best compromise between simplicity and relevance for clinicians. It can portray the evolution over time of most clinically relevant variables, such as mean arterial pressure (MAP), heart rate (HR), cardiac output (CO), and central venous pressure (CVP).

In particular, the hemodynamic responses simulated by this model match with physiological expectations during both volume loss (e.g., hemorrhage) and intravenous therapy (e.g., fluid resuscitation).

As a model for predicting hemorrhagic shock in emergency settings, which lend themselves to pitfalls in physiological accuracy and interpretability, our aim is to create a tool that is both reliable and simple-to-use.

Therefore, we opted for the Zenker model, as a starting point; however, we introduce an upgrade, dubbed the ZenCur model (i.e., combining Zenker and Curcio) that permits a more realistic elucidation of the pathophysiological mechanisms of shock.

The rationale for the modification to the Zenker model, qualitatively correct in its response to variations in hydration status alone (total intravascular volume) throughout the bleeding phase, was its failure to predict the subsequent post-bleeding phase, since it is devoid of a fundamental homeostatic mechanism triggered by hemorrhage [13].

A first key modification of Zenker's model involved incorporating the homeostatic mechanism of transcapillary refill that accompanies significant degrees of fluid/blood loss.

Although a new theory, i.e., the Revised Starling Principle, has emerged over the past decade [14–16], it was nevertheless deemed necessary to incorporate the effects of transcapillary fluid exchange into our model, based on more established understanding of physiology, as echoed by several authors [13, 17–24].

For example, Prist et al. [21] demonstrated that the degree of hypovolemia resulting from the same total overall blood loss varied significantly at different rates of loss. At slower rates, compensation was greater due to capillary refill. Instead, the higher the rate, the more hemodynamic deterioration tended to be precipitous. These results warranted the inclusion of transcapillary fluid exchange as a key determinant that provided the rationale for its incorporation into our computational model.

In particular, as an adjunct to the term which represents a withdrawal component due to the hemorrhage, we consider another complementary term representing transcapillary refill. The latter is clinically relevant inasmuch as it represents a non-negligible response of the circulatory system to any significant degree of acute blood loss. It also allows our model to realistically predict the trend of MAP, CO, and HR for a few hours after the end of the bleeding. It also allows to establish whether the patient needs an urgent transfusion to survive.

Furthermore, in the graphs plotting the hemodynamic variables in the first instants of the simulations (i.e., state of the subject prior to hemorrhage), the Zenker model presents a “ripple” not attributable to physiological conditions.

Therefore, to correct for it, we also introduced mathematical constraints into the initial conditions so as to eliminate the transient (“ripple”) in the curves at the beginning of the simulation. This transient effect occurs prior to achieving steady-state conditions for several physiological variables.

In synthesis, we seek to understand the extent to which a simple cardiovascular model can replicate the known pathophysiological responses of healthy swine subjected to severe hemorrhage and whether such a model can elucidate the cause-effect relationships that underlie the predictable pattern of behaviors.

In this article, we will describe the ZenCur model in greater detail and discuss the results achieved by comparing simulation and *in vivo* data.

The main purpose of this work is to demonstrate the value of our mathematical model in the analysis of experimental behavior, using four specific laboratory animals.

## 2. Materials and Methods

Zenker's cardiovascular system model consists of a single-ventricle heart that pumps blood into the large systemic arteries, equated to linear capacitors (in accord with the Windkessel model), whereby arterial pressure is governed by feedback control mechanisms [25, 26]. The pulmonary circulation is excluded for simplicity.

Thus, Zenker developed this ordinary differential equation (ODE) model of the left heart, characterized by a single-cycle systolic phase and a diastolic phase.

His simplified 5-ODE model of the cardiovascular system was inclusive of baroreflex pressure control, with a specific focus on the role of cardiac contractility, peripheral resistance, and blood volume, as well as the interactions among these factors [12].

The model proposed by Zenker et al. represented a landmark innovation inasmuch as it represented a shift from beat-by-beat cardiac dynamics to a continuous variation of beat characteristics. As such, the Zenker model efficiently simulated physiological feedback loops as a continuous temporal representation.

By contrast, previous authors [27] typically based their cardiovascular simulations on the computationally demanding constraints of intra-beat dynamics.

More than to simulation scenarios requiring the specifics of the single cardiac cycle, the Zenker model is rather attuned to modeling whose focus is the overarching pattern of inter-beat dynamics, calibrated to a continuous, clinically significant, timescale.

The ZenCur model not only contains every feature of Zenker's model but also includes the adjunct homeostatic mechanism of transcapillary refill.

**Figure 1:**
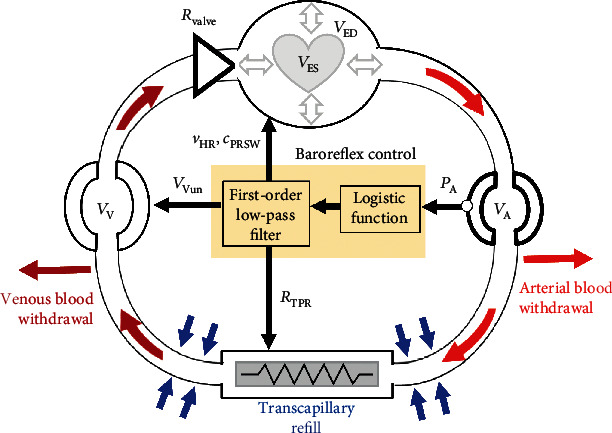
Schema of the ZenCur model. At the top center, the pulsing univentricular heart goes from end-diastolic *V*_ED_ to end-systolic volume *V*_ES_. Via a system of valves, the blood is driven (clockwise in the figure) into the arterial compartment. The pressure generated must overcome the total peripheral resistance *R*_TPR_ and guarantees blood flow to the venous compartment which feeds back into the heart. Variations from the set point of arterial pressure *P*_A_ determine corresponding variations in actuators. These include heart rate *ν*_HR_, *R*_TPR_, myocardial contractility *c*_PRSW_, and unstressed venous volume *V*_Vun_. Of note: variations of *P*_A_ are processed through a sigmoidal nonlinearity and a linear element with first-order low-pass filter characteristics. Our model accounts for bleeding and for the physiological compensatory mechanism called transcapillary refill (adapted from Figure 2 in Zenker S, Rubin J, Clermont G., From Inverse Problems in Mathematical Physiology to Quantitative Differential Diagnoses, *PLoS Computational Biology*, 3 (2007), 2074).

In Subsections 2.1 and 2.2, we will describe the circulatory and control systems of the Zenker model [12], respectively.  Figure 1 shows a schematic representation of the model.

### 2.1. Circulatory Model

The foremost role of the heart is to pump blood through the circulatory system, ensuring the transport of fluid, gases, and nutrients throughout the entire system. In the cardiocirculatory model considered, the pump consists of the left chambers of the heart (i.e., atrium plus ventricle).

Via a two-phase process, every cardiac cycle ejects a volume of blood into the arterial tree then relaxes. In the first phase, or systole, the ventricle contracts so a fraction of its blood is forcefully ejected through the aortic (outflow) valve into the aorta, once the intraventricular pressure surpasses the aortic pressure. Prerequisite mitral (inflow) valve closure assures unidirectional flow. During the second phase, or diastole, a relatively longer period of ventricular relaxation ensues. The abrupt drop in intraventricular pressure causes the aortic valves to close and the mitral valve to open, such that the ventricle fills with blood.

The net volume of blood ejected into the aorta by each systolic contraction is the stroke volume, calculated as follows:

(1) $$\begin{array}{c} V_{\text{S}}=V_{\text{ED}}-V_{\text{ES}}, \end{array}$$

in which the stroke volume (*V*_S_) is the difference between the end-diastolic volume (*V*_ED_), when the ventricle is most replete with blood, and the end-systolic volume (*V*_ES_) after the cardiac contraction, but immediately prior to ventricular relaxation.

Assuming that ventricular volume will not usually decrease below *k*_*V*ED0_, we can define ${\tilde{V}}_{\text{ES}}$, i.e., the current end-systolic volume, as a function of the end-diastolic volume, as follows [12]:

(2) $$\begin{array}{c} {\tilde{V}}_{\text{ES}}\left(V_{\text{ED}}\right)=\left\{\begin{array}{cc} \max\left(k_{\text{VED}0},V_{\text{ED}}-\frac{C_{\text{PRSW}}\cdot \left(V_{\text{ED}}-k_{\text{VED}0}\right)}{P_{\text{A}}-P_{\text{ED}}}\right), & \text{if }P_{\text{A}}>P_{\text{LV}}\left(V_{\text{ED}}\right)=P_{\text{ED}},\\ k_{\text{VED}0}, & \text{otherwise}, \end{array}\right. \end{array}$$

where *k*_VED0_ is the volume at which intraventricular pressure equals 0 mmHg [28], *P*_LV_ is the ventricular pressure, *C*_PRSW_ represents the linear relation between stroke work and end-diastolic volume [29], *P*_A_ is the arterial pressure, and *P*_ED_ is end-diastolic pressure within the ventricle, respectively.

The current end-diastolic volume ${\tilde{V}}_{\text{ED}}$ as a function of *V*_ES_ can be expressed as follows [12]:

(3) $$\begin{array}{c} {\tilde{V}}_{\text{ED}}\left(V_{\text{ES}}\right)=\left\{\begin{array}{cc} -\frac{1}{k_{\text{ELV}}}\cdot \log\left(-\frac{P_{0\text{LV}}}{P_{\text{CVP}}+P_{0\text{LV}}}\cdot e^{-k_{\text{ELV}}\cdot k_{\text{VED}0}}\cdot \left(e^{-k_{\text{ELV}}\cdot \left(\left(P_{\text{CVP}}+P_{0\text{LV}}\right)/R_{\text{valve}}\right)\cdot t_{\text{Diast}}}-1\right)++e^{-k_{\text{ELV}}\cdot \left(V_{\text{ES}}+\left(\left(P_{\text{CVP}}+P_{0\text{LV}}\right)/R_{\text{valve}}\right)\cdot t_{\text{Diast}}\right)}\right), & \text{if }P_{\text{CVP}}>P_{\text{LV}}\left(V_{\text{ES}}\right)=P_{\text{ES}},\\ V_{\text{ES}}, & \text{otherwise}, \end{array}\right. \end{array}$$

where *k*_ELV_ is a constant characterizing the passive empirical pressure/volume relationship [28], *P*_0LV_ is the passive empirical ventricular pressure [28], *P*_CVP_ is the central venous pressure, and *P*_ES_ is the ventricular pressure at end-systolic volume, respectively. In particular, *t*_Diast_ is the duration of diastole:

(4) $$\begin{array}{c} t_{\text{Diast}}=\frac{1}{\nu_{\text{HR}}}-t_{\text{Syst}}, \end{array}$$

where

(5) $$\begin{array}{c} t_{\text{Syst}}=\frac{1}{\nu_{\text{HR}0}\max}\cdot 0.8, \end{array}$$

where *ν*_HR_ is the heart rate and *ν*_HR0_max is the maximum initial value for heart rate.

The end-systolic ventricular volume *V*_ES_ is evaluated as [12]

(6) $$\begin{array}{c} \frac{dV_{\text{ES}}}{dt}=\left({\tilde{V}}_{\text{ES}}-V_{\text{ES}}\right)\cdot \nu_{\text{HR}}. \end{array}$$

The end-diastolic ventricular volume *V*_ED_ is described by the following differential equation [12]:

(7) $$\begin{array}{c} \frac{dV_{\text{ED}}}{dt}=\left({\tilde{V}}_{\text{ED}}-V_{\text{ED}}\right)\cdot \nu_{\text{HR}}. \end{array}$$

A simple Windkessel model is used to represent systemic circulation, comprising linear compliances *C* in lieu of the large-vessel arteries (of volume *V*_A_) and veins (of volume *V*_V_), with their respective pressures, according to the following equation:

(8) $$\begin{array}{c} P_{\beta }=\frac{V_{\beta }-V_{\beta \text{un}}}{C_{\beta }}, \end{array}$$

where *β* is “A” or “V”; *P*_V_=*P*_CVP_; *V*_*β*un_ is the corresponding unstressed volume of “A” or “B” (defined as the nonzero volume whose pressure equals 0 mmHg); *C*_*β*_ are the compliances.

The pressure difference is the numerator of the equation connecting the arterial and venous compartments through a linear resistor, while the denominator is the total peripheral resistance *R*_TPR_. This equation regulates arterio-venous capillary blood flow, i.e., the total volume of blood traversing the entire systemic capillary bed per minute, expressed as follows:

(9) $$\begin{array}{c} I_{\text{C}}=\frac{P_{\text{A}}-P_{\text{CVP}}}{R_{\text{TPR}}}. \end{array}$$

The veno-arterial flow (i.e., cardiac output) *I*_CO_ represents the total flow generated by the heart per unit time, given by the product of heart rate *ν*_HR_ and stroke volume *V*_S_. Equation (1) allows us to express it as follows:

(10) $$\begin{array}{c} I_{\text{CO}}=\left(V_{\text{ED}}-V_{\text{ES}}\right)\cdot \nu_{\text{HR}}. \end{array}$$

Considering conservation of mass (blood volume) at the nodes of a circuit mesh, the arterial and venous volumes over time may be described according to the following differential equations [12]:

(11) $$\begin{array}{c} \frac{{dV}_{\text{A}}}{dt}=I_{\text{CO}}-I_{\text{C}}, \end{array}$$

(12) $$\begin{array}{c} \frac{{dV}_{\text{V}}}{dt}=-\frac{{dV}_{\text{A}}}{d\text{t}}+I_{\text{external}}=-\frac{{dV}_{\text{A}}}{dt}+I_{\text{bleed}}+I_{\text{refill}}=I_{\text{C}}-I_{\text{CO}}+I_{\text{bleed}}+I_{\text{refill}}, \end{array}$$

where *I*_external_ represents the sum of the two terms, *I*_bleed_ and *I*_refill_. In the case of a bleeding vein, for example, the former is the external bleeding rate, whereas the latter is the rate of transcapillary refill (i.e., interstitial fluid migration to the venous compartment).

The essential difference between Zenker's model and ours is that the former lacks any notion of transcapillary refill: this compensatory mechanism commences at the onset of the bleeding and continues until homeostasis is achieved.

Notably, blood volume after blood loss is replenished primarily by way of a transcapillary refill. Being a complex homeostatic process, a host of interrelated pathophysiological pathways is triggered. In sum, fluid reserves within the extravascular compartment are mobilized and redistributed in response to hemorrhage. Of note, fluid already present within the body is thus promptly deployed and relocated to correct acute volume deficits of the intravascular compartment [19]. These fluid exchanges occur at the level of the capillary walls as first elucidated by Starling [30].

Therefore, we deemed including this key homeostatic mechanism into our model both warranted and necessary. In fact, during severe hemorrhage, its role is anything but negligible. Accordingly, it was included among the key hemodynamic variables at play during hemorrhage so as to provide a more accurate representation to model reality. In our discussion, we have considered only “simple” bleeding (i.e., with minimal injury to tissues).

The basis of our modeling of transcapillary refill derives from the pressure differential between central venous and interstitial fluid pressures. The latter is assumed constant and equal to the steady-state *P*_CVP_ value (*P*_CVP0_):

(13) $$\begin{array}{c} I_{\text{refill}}=k_{\text{Vcap}}\cdot \left(P_{\text{CVP}0}-P_{\text{CVP}}\right), \end{array}$$

where *k*_Vcap_ is the proportionality constant between the rate of transcapillary and the venous-to-interstitial pressure difference.

### 2.2. Control Model

In the context of cardiovascular homeostasis, baroreflex control of blood pressure is based on the consensus representation of central processing of baroreceptor sensory input, i.e., the combination of a sigmoidal nonlinearity (logistic function) and a linear system [31, 32].

Instead of relying on a more complex, albeit physiologically more accurate, interplay of sympathetic and parasympathetic (inhibitory) components, for the sake of simplicity, Zenker et al. reduced baroreflex activity to a net sympathetic (activating) output. Zenker's single-ventricle model of the heart considers timescales that are orders of magnitude higher than a single beat. Consequently, the linear portion of the baroreflex feedback loop is simplified to display first-order low-pass characteristics, with a time constant commensurate to the least rapid actuator response (i.e., unstressed venous volume control) [12]. Delays attributable to the transmission of baroreflex signaling along neuronal pathways are considered null. Based on these assumptions, the features characterizing stimulatory output of baroreflex central processing, over time, are governed by the following differential equation [12]:

(14) $$\begin{array}{c} \frac{dS_{E}}{dt}=2\cdot \pi \cdot \nu_{\text{cut}}\cdot \left(S-S_{E}\right), \end{array}$$

where the parameter *ν*_cut_ represents the first-order low-pass cut-off frequency (determining the time constant of the baroreflex response) and *S* is the sympathetic nervous activity.

The stimulating output *S*_*E*_(*t*) of the feedback loop affects heart rate (${\nu \hat{I}½}_{\text{HR}}$), total peripheral systemic vascular hydraulic resistance (*R*_TPR_), myocardial contractility (*C*_PRSW_), and unstressed venous volume (*V*_Vun_) to realign blood pressure in response to deviations from the set point, based on the linear transformations below [12]:

(15) $$\begin{array}{c} \gamma =S_{E}\cdot \left(\gamma_{\max}-\gamma_{\min}\right)+\gamma_{\min}, \end{array}$$

where *γ* = *ν*_HR_, *R*_TPR_, or *C*_PRSW_, and

(16) $$\begin{array}{c} V_{\text{Vun}}=\left(1-S_{E}\right)\cdot \left(V_{\text{Vun}}\max-V_{\text{Vun}}\min\right)+V_{\text{Vun}}\min. \end{array}$$

The formula of Equation (16), in particular, reflects the fact that the venous capacitance vessels contract in response to decreasing blood pressure, thus reducing their unstressed volume.

By combining Equations (6), (7), (11), (12), and (14), then including the dependencies relevant to the coupling of the system as explicit terms, we obtain Zenker's five ODE system [12]:

(17) $$\begin{array}{c} \frac{dV_{\text{ES}}}{dt}=\left({\tilde{V}}_{\text{ES}}\left(V_{\text{ED}},V_{\text{A}},S_{E}\right)-V_{\text{ES}}\right)\cdot \nu_{\text{HR}}\left(S_{E}\right), \end{array}$$

(18) $$\begin{array}{c} \frac{dV_{\text{ED}}}{dt}=\left({\tilde{V}}_{\text{ED}}\left(V_{\text{ES}},V_{\text{V}}\right)-V_{\text{ED}}\cdot \nu_{\text{HR}}\left(S_{E}\right)\right., \end{array}$$

(19) $$\begin{array}{c} \frac{dV_{\text{A}}}{dt}=\frac{P_{\text{A}}\left(V_{\text{A}}\right)-P_{\text{V}}\left(V_{\text{V}},S_{E}\right)}{R_{\text{TPR}}\left(S_{E}\right)}-\left(V_{\text{ED}}-V_{\text{ES}}\right)\cdot \nu_{\text{HR}}\left(S_{E}\right), \end{array}$$

(20) $$\begin{array}{c} \frac{dV_{\text{V}}}{dt}=-\frac{dV_{\text{A}}}{dt}+I_{\text{external}}\left(t\right), \end{array}$$

(21) $$\begin{array}{c} \frac{dS_{E}}{dt}=2\cdot \pi \cdot \nu_{\text{cut}}\cdot \left(S-S_{E}\right). \end{array}$$

The stimulatory output *S*_*E*_ is the pivotal control mechanism of homeostasis for the cardiovascular system. It links the individual components together, whereas the coupling between the equations describing cardiac and circulatory activities reflects the cyclical nature of cardiac and circulatory functioning.

The dynamics of the system are obtained by solving a system of five ordinary differential equations (17–21). The full list of equations and initial conditions is provided in “Appendix.” Table 1 summarizes all the model variables.

The model was implemented in C++ (Microsoft Visual Studio 2017 Community Edition), MATLAB (Mathworks MATLAB 2009b), and PHP, using a fixed-step, fourth-order Runge-Kutta numerical integration scheme [33], and is available for simulation on the CNR IASI BioMatLab web platform: http://biomatlab.iasi.cnr.it/models/login.php.

### 2.3. Model Parameters

All model parameters and their meanings are summarized in Table 2, whereas Table 3 shows the calibrated parameter sets. In Table 3, the parameters marked with the symbol “†” are constants, whose experimental values are taken from Glower et al. [28]. The parameters marked with “‡” indicate those parameters whose values depend on the physical characteristics of the laboratory animal (swine) considered, while those marked with “∘” are the parameters characterized by the conditions in which the bleeding occurred in the laboratory. The parameters set by the user have this distinctive symbol “⋄,” while the parameters with “★” are the parameters whose values were set to steady-state conditions as the initial conditions (details are shown in the appendix). Finally, the free parameters are indicated with the “∗” symbol. The physiological reference values for the free parameters were taken from Zenker et al. [12].

Parameters have been estimated through model calibration: specifically, calibration of the vector of target parameters *θ* was conducted via an algorithm that seeks to minimize loss function (cost function).

(22) $$\begin{array}{c} \theta \in \left\{R_{\text{valve}},C_{A},C_{V},V_{\text{Aun}},\nu_{\text{cut}},S_{\text{sat}},P_{\text{Aset}},\nu_{\text{HR}}\min,\nu_{\text{HR}}\max,C_{\text{PRSW}}\min,C_{\text{PRSW}}\max,R_{\text{TPR}}\min,R_{\text{TPR}}\max,V_{\text{Vun}}\min,V_{\text{Vun}}\max,k_{\text{Vcap}}\right\}. \end{array}$$

Optimization was performed using the nonlinear programming solver fminsearch in MATLAB so as to determine minimum values for this objective function:

(23) $$\begin{array}{c} J\left(\theta \right)=\min\overset{N}{\underset{t=1}{\sum }}{\left(\frac{P_{\text{A }\exp}\left(t\right)-{\overset{\wedge }{P}}_{\text{A sim}}\left(t,\theta \right)}{P_{\text{A }\exp}\left(t\right)}\right)}^{2}+{\left(\frac{\nu_{\text{HR }\exp}\left(t\right)-{\overset{\wedge }{\nu }}_{\text{HR sim}}\left(t,\theta \right)}{\nu_{\text{HR }\exp}\left(t\right)}\right)}^{2}+{\left(\frac{I_{\text{CO }\exp}\left(t\right)-{\overset{\wedge }{I}}_{\text{CO sim}}\left(t,\theta \right)}{I_{\text{CO }\exp}\left(t\right)}\right)}^{2}, \end{array}$$

where *X*_exp_(t) is the measurement of the observed variable at time *t* and the hat symbol indicates the corresponding value simulated by the model, while *N* is the number of observations over which the normalized sum of squares was computed. The lower the *J*(*θ*) value, the better the model performance.

Sensitivity values of our parameter estimates were calculated, in line with Beard et al. in [34]. Specifically, we computed the relative change in the mean-square error difference between data and model simulations associated with a variation in the parameter value.

Assuming that *E* is the minimum normalized mean-squared error difference between model prediction and data, then the sensitivity associated with parameter *ξ* is estimated as

(24) $$\begin{array}{c} S_{\theta }\;\left(\xi \right)\approx \frac{E\left(\xi +0.1\xi \right)-E\left(\xi \right)}{0.1}. \end{array}$$

**Figure 2:**
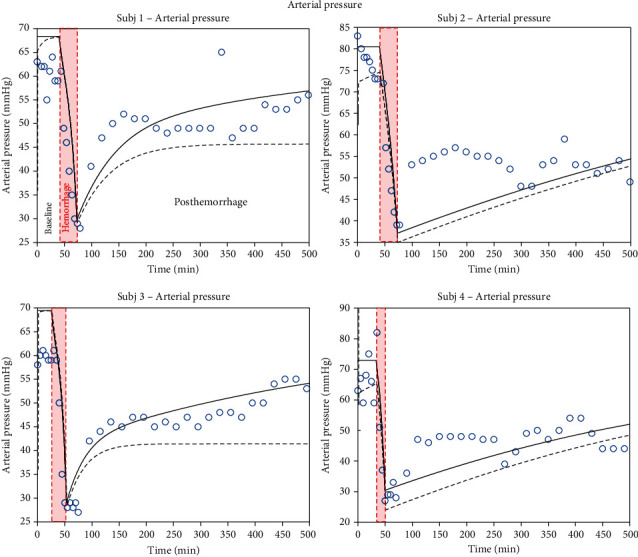
Time course of arterial pressure. Model-simulated evolution of arterial pressure for 4 different subjects: the continuous black line represents the ZenCur model forecast; the black dashed line represents the Zenker model forecast, while the blue circles represent the experimental data.

**Figure 3:**
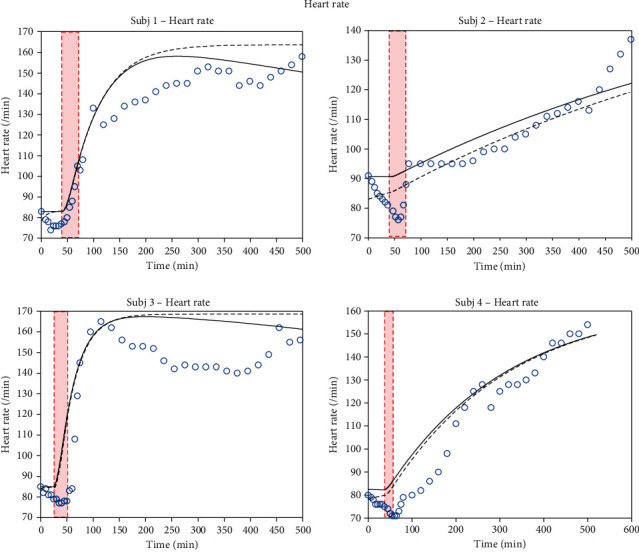
Time course of the heart rate. Model-simulated evolution of the heart rate for 4 different subjects: the continuous black line represents the ZenCur model forecast; the black dashed line represents the Zenker model forecast, while the blue circles represent the experimental data.

**Figure 4:**
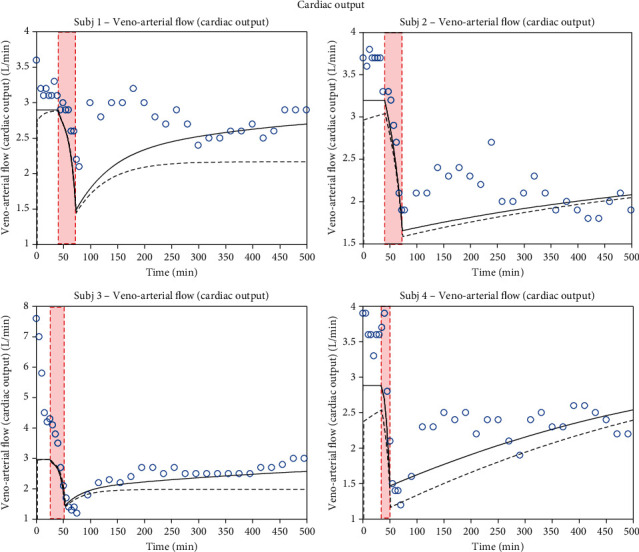
Time course of cardiac output. Model-simulated evolution of cardiac output for 4 different subjects: the continuous black line represents the ZenCur model forecast; the black dashed line represents the Zenker model forecast, while the blue circles represent the experimental data.

Based on the data in Figures 2–4, sensitivities for all parameters identified are listed in Table 4. Computed sensitivity values range from a minimum of 0.002 (for *R*_valve_) to 3.656 (for *V*_Vun_Min).

In our model, as shown in Table 4, the parameters with the highest sensitivity rankings are *V*_Vun_Min and *V*_Vun_Max: during a hemorrhage (and especially after a hemorrhage without resuscitation), the volume of unstressed venous blood available is the preponderant issue. Indeed, at any given moment, approximately 70% of the systemic blood volume is flowing through veins [35].

Accordingly, cardiac output is largely dependent on venous return. This venous reserve plays a major role in maintaining venous pressure during acute volume deficits. Two factors are credited for the venous system ability to compensate for acute hemorrhage: mobilization of unstressed blood volume and changes in venous capacitance *C*_V_ [36]. In fact, conversion of unstressed blood volume to stressed blood volume is accompanied by significant reductions in the capacitance of venous vessels throughout the circulatory system [36].

In terms of preserving venous pressure, mobilization of unstressed blood volume prevails as the more effective mechanism. During acute hemorrhage of any significance, adequacy of mean filling pressures bolsters venous return and thereby cardiac output [36].

Furthermore, hypovolemia prompts immediate activation of baroreceptors located at the level of the carotid artery, aortic arch, left atrium, and pulmonary veins. This activation triggers a reflex arc, resulting in reduced vagal tone, as well as a corresponding increase in norepinephrine release.

At the arteriolar level, norepinephrine increases contraction at the precapillary sphincters (mainly of the splanchnic and musculoskeletal vessels) producing a consensual increase in the total peripheral resistance *R*_TPR_max. This, in turn, is the key factor in maintaining blood pressure constant with respect to reference values (*P*_Aset_).

At the cardiac level, the reduction of parasympathetic tone and the increased release of norepinephrine produce tachycardia. In fact, even the *ν*_HR_max parameter has a noteworthy sensitivity in our model.

### 2.4. Experimental Hemorrhagic Shock Protocol

The experimental protocol of hemorrhagic shock in the swine model was conducted in the Institute for Research in Military Medicine (IRMM) of the Faculty of Medicine of the Hebrew University of Jerusalem, Israel, and the Israel Defense Forces Medical Corps. A total of 4 laboratory animals were used for the development of the current model. In Table 5, the mean baseline characteristics of the animals are shown.

The animals were fed with standard laboratory chow and kept adequately hydrated with free access to water. Twelve hours before the operative procedures, the animals were deprived of solid food, but their access to water was not limited. The experimental protocol was acute: the animals were bled (35% of total blood volume) and then monitored for up to 7 hours without resuscitation.

All animals were euthanized immediately after the operative procedure, strictly applying the international laws for treating animals in experimental model protocols (Guide for the Care and Use of Laboratory Animals, National Academy Press, Washington, D.C. 1996). Animal care and experimental procedures were approved by the Ethics Committee of the Faculty of Medicine of the Hebrew University of Jerusalem, Israel (MD-13-13751-3).

The experiments were performed with the assistance of experienced veterinarians.

### 2.5. Anesthesia, Maintenance, and Materials

Swine were sedated with xylazine (1 mg/kg, IM, Eurovet Animal Health B.V., Bladel, Netherlands) and anesthesia induced with ketamine (10 mg/kg, IM, Vétoquinol S.A., Lure, France). The ear vein was then cannulated for intravenous administration of a mixture of diazepam (2 mg, IV, TEVA Pharmaceutical Industries Ltd., Jerusalem, Israel), ketamine (400 mg, IV), propofol (1-4 mg/kg, IV, Fresenius Kabi Austria Gmbh, Linz, Austria), and tramadol (5 mg/kg, IM, Rafa Laboratories 294td., Jerusalem, Israel) for analgesia. Cefazolin (1 g, IV, Panpharma S.A., Luitré, France) was given as prophylactic measure. The pigs were then intubated with a cuffed silastic endotracheal tube (7.0 mm, Portex Tracheal Tube, Kent, UK).

Anesthesia was maintained with 2% isoflurane (Piramal Critical Care Inc., Bethlehem, PA, USA) in 100% oxygen, and animals were ventilated using controlled mechanical ventilation (Excel 210-SE anesthesia machine m-Datex-Ohmeda Inc., Madison, WI, USA, or Narkomed-2B Anesthesia Machine—North American Drager, Houston, TX, USA). Tidal volume was set to 10 mL/kg with a respiratory rate of 13-15 breaths per minute, adjusted to an end-tidal CO_2_ (ETCO_2_) of 35 mmHg at baseline.

### 2.6. Surgical Protocol

Each animal was placed in the supine position on the operating table which was covered by warmed blankets and mattress in order to maintain the temperature of each animal at 39°C (±0.5°C).

The following vessels were cannulated using a catheter-over-wire technique (Seldinger) with introducers inserted into the vessel (Cordis, Fremont, CA, USA): the left common carotid artery for invasive blood pressure monitoring and heart rate; the right internal jugular vein for venous blood sampling; pulmonary artery catheter placement (Swan-Ganz CCOmbo, Edwards Lifesciences, Irvine, CA, USA); the right femoral artery for arterial blood sampling and induction of bleeding. A Tiemann Catheter 12F was placed for collection of urine. Body temperature was monitored rectally. A pulse oximeter was placed on the tongue to measure oxygen saturation. Continuous three-lead ECG monitoring (Marquette, GE Healthcare, Chicago, USA) was obtained using electrodes placed on the animal's right forelimb, left forelimb, and left hind limb. At the end of the observation period, surviving animals were euthanized by an intravenous injection of KCl solution (Fagron Group BV, Rotterdam, Netherlands). Before bleeding, each animal was weighed to calculate the total circulating blood volume. The amount of blood to be withdrawn represents 30-35% of the estimated blood volume [37].

Controlled bleeds, as empirical replications of these complex biological processes, are performed manually by drawing 50 mL aliquots of blood from the right femoral artery via a syringe.

The rate of bleeding was monitored continuously, so as to maintain mean arterial pressures (MAP) above 30 mmHg. Whenever MAP dropped below that threshold, blood loss was stopped in order to allow the animal to recover prior to resumption of bleeding.

The controlled bleeding was then resumed, filling as many 50 mL syringes as needed until the target volume was reached.

One should bear in mind that bleeding is not a linear process. In fact, initially, the rate of hemorrhage is much faster but then tends to wane over time, reflecting the dynamics of an actual trauma with hemorrhage. In the moments following an injury, blood loss is more profuse until a tamponade, vasoconstriction, and other hemostatic mechanisms set in to curb the hemorrhage.

The bleeding rate is variable during the procedure: very high at the beginning and gradually diminish. In fact, the bleeding does not have a fixed duration but it varies between 30 and 60 minutes.

## 3. Results

An accurate, reliable mathematical model, calibrated using animal studies, would be of great benefit for the assessment of the likelihood of patient survival in relation to the severity of the traumatic insult and hence provide valuable information regarding the urgency of transport for definitive care.

We determined our approach to simulating the complex responses of the cardiovascular system to hemorrhage after a comprehensive literature search and evaluation of existing mathematical modeling techniques relevant to the cardiovascular domain. Our approach was to first determine the hemodynamic effects of bleeding in the absence of fluid resuscitation and then establish a reference time range for survival prior to massive transfusion.

The current model can be characterized as a simplified, ordinary differential-equation model of the cardiovascular system. As such, it can be utilized to quantitatively analyze and simulate systemic responses to clinically significant variations in blood volume and intravascular fluids typically occurring during hemorrhage. The rationale and design of the model were based on pursuing an acceptable trade-off between complexity (i.e., physiological fidelity) and alignment with the empirical data. The parameters of the model were calibrated based on hemodynamic measurements in experimental animal studies. The model fit in the 4 test animals and simulations conducted thus far have proven the model to also perform correctly, in qualitative terms, with regard to the targeted, clinically relevant, physiological responses of three hemodynamic variables simultaneously.

Now, we describe the evolution of key physiological variables comparing the ZenCur model to that of Zenker: mean arterial pressure *P*_A_, heart rate *ν*_HR_, and cardiac output *I*_CO_ that are depicted in Figures 2–4, respectively. In this figure, the data refer to 4 separate laboratory swine (subjects 1 to 4), which underwent controlled hemorrhage. The black continuous curves plot the time course of the ZenCur model forecast. The black dashed curves plot the time course of the Zenker model forecast. The blue circles are the corresponding experimental values.

The curves of the predictive model were determined by parameter calibration for each animal. The calibrated parameters are summarized in Table 3 (for the Zenker model, as there is no transcapillary refill mechanism, the *k*_Vcap_ parameter is set to zero). Clearly, with the reported values of these parameters, the model equations reflected all forecasted time courses simultaneously. We can distinguish three defined areas in the diagram: the baseline prior to hemorrhage, hemorrhage, and post-hemorrhage phases (the duration of hemorrhage corresponds to the red-shaded area). The total duration of the experiments for each animal was about 500 minutes.

Taking into consideration Figure 2, relative to arterial pressure, we note that as soon as bleeding begins, the curves of both models fall rapidly and then rise again with a progressively accentuated slope when bleeding is interrupted. We can see that the curves of the Zenker model have a less pronounced slope in the post-bleeding phase. During bleeding, the forecast from the models and the experimental data are in agreement for all 4 subjects under examination. However, the fit of the ZenCur model is best during the post-hemorrhage phase in subjects 1 and 3, which show a recovery of blood pressure values that mirror the experimental data rather closely. On the contrary, in subjects 2 and 4, despite a careful calibration of the parameters, the model drifts away from the time course of variable *P*_A_. The latter presents a rather irregular behavior in these subjects with respect to these 2 swine.

The temporal evolution of the *ν*_HR_ heart rate for the 4 subjects under examination (Figure 3) shows how the response to hemorrhage can follow two different patterns: one characterizing subjects 1 and 3 and the other, subjects 2 and 4. The models follow both patterns. In particular, the patterns for swine 1 and 3 show a sudden rise at the beginning of the bleeding and then drop after bleeding. In swine 2 and 4, the time courses of heart rate have a linear evolution increasing over time after the onset of hemorrhage. In these two subjects, the curves do not rise suddenly, but rather gradually, up to the end of the period of observation.

The last physiological variable for which we compare the predictions of the two models and observations is cardiac output (*I*_CO_, Figure 4). The trend is similar to that seen for arterial pressure. Throughout the hemorrhage, there is a rapid drop in CO, both for theoretical predictions and the experimental data. At the end of the hemorrhage, there is an absolute minimum point followed by recovery for subjects 1 and 3 that stabilizes around 3 L/min. Instead, for the other 2 animals (2 and 4), we have, at first, an immediate rise in the curve followed by a slow decrease starting from minute 400.

We observe that the CO curves of the ZenCur model for the 4 subjects fit the experimental data with greater precision than the curves of the Zenker model.

As shown in almost every graph plotting hemodynamic variable data, there is a margin of deviation between simulation values (solid black line) and the corresponding experimental values that all fall within the initial baseline time intervals.

This is attributable to an irregular fluctuation of the experimental data at the beginning of the sample survey since it is assumed that the person to be hemorrhaged maintains steady-state hemodynamic values before blood sampling (in accordance with our simulation).

Unfortunately, this difference between the hemodynamic values simulated by the model and the experimental data is heavily reflected on the value of the cost function which undergoes an unjustified increase in the value.

The values of the cost function *J* are shown at the bottom of Table 3, where *J*_nobase_ represents the value of *J* calculated in the absence of the experimental points of the baseline. The greatest difference between the two values is found in the case of subject 3.

## 4. Discussion

When compared to the experimental data in our possession, our model predicts, fairly well, the time courses of the hemodynamic variables considered in each of the three phases that characterize the hemorrhagic event: baseline, hemorrhagic, and post-hemorrhagic.

Despite existing limitations (such as the confounding effects of anesthesia, an anything but complete representation of baroreflex control, and relatively small number of subjects), we believe it, nevertheless, captures many of the main features of acute hemorrhage and may provide new insights into the complex interactions of the process.

We deem these results partly due to the introduction of the homeostatic mechanism of transcapillary refill, which encapsulates the innovation of our model with respect to Zenker's. By integrating this compensatory mechanism into our model, we simulated the temporal trend of all hemodynamic variables with greater precision and, in particular, significantly improved forecasting regarding the post-hemorrhagic phase: without taking transcapillary refill into account, Zenker's model generated unreliable predictions in the post-hemorrhagic phase, thus justifying the change.

According to classical theory, the mechanism of transcapillary refill, which governs the net movement of fluid and extracellular protein from the interstitial compartment into the intravascular compartment, is described by the Starling equation [19].

However, as an evolution of the original concept by Hu and Weinbaum [38], Levick and Michel [14] subsequently proposed the so-called “Revised Starling Principle” (RSP).

The Starling principle explains the movement of fluid between blood and interstitia as a result of hydrostatic- and colloid-osmotic (oncotic) pressure differentials across the capillary membrane [15]. In contrast, the RSP recognizes that a balance of pressures cannot halt fluid exchange, given that microvessels are semipermeable to macromolecules. As such, true Starling equilibria cannot occur, but rather steady states are maintained at some finite, albeit low, level of filtration within most tissues [15].

The traditional Starling principle thus needed revision due to insights into the role of interstitial fluid (ISF) pressures, also acknowledging the functional properties of the endothelial glycocalyx as a semipermeable barrier [14].

The revised Starling equation and the glycocalyx model represent an improved paradigm in the management of intravenous fluid therapy in clinical situations, according to various scholars [39, 40].

For other scholars [16], however, the hypothesis still deserves thorough clinical validation. In particular, these authors argue against “the Extension of Starling's Principle” [15], based on the purported role of “*the endothelial glycocalyx layer, which moves the oncotic gradient from being between the plasma and the interstitium to between the plasma and a virtually protein-free space between the glycocalyx and the endothelial cell membrane, which dramatically changes the prerequisites for fluid absorption from tissue to plasma [16].*”

According to Hahn et al. [16], moreover, many experimental and clinical observations in humans fail to fully support these novel proposals for microcirculation. The key issue concerns the role of the glycocalyx in the Revised Starling Principle in terms of effective reabsorption of fluid, on the part of skeletal muscle, when capillary filtration pressure acutely diminishes.

Therefore, the homeostatic mechanism of transcapillary refill remains controversial and the RSP appears weak (not only in the opinion of Hahn et al. [16], but ours as well) since it essentially concerns steady-state conditions: the cardiovascular system, as a complex system, is arguably dynamic nonetheless [16].

Flow motion via vasomotion is a phenomenon that occurs instant by instant, through a dynamic equilibrium, and therefore must be duly considered in order to understand clinical observations in humans [16].

Given the dynamic nature of vasomotion, we deem the classic Starling principle more accurate and, thus, opted to adopt it for our model. Furthermore, in the light of the results obtained by comparing the predictions of our model with experimental data, we consider the transcapillary refill (even if modeled primarily by hydraulic pressure differences) an important and non-negligible element.

Moreover, one should bear in mind that, due to compensatory mechanisms, assessing bleeding patients is challenging because current clinical tools lack sensitivity in that they measure vital signs that remain stable during the early stages of bleeding. Consequently, there is a need to understand and assess whole systems that incorporate compensatory mechanisms for reduced circulating blood volume and how these interact over time with ongoing hemorrhage [41].

More accurate predictions of the temporal evolution in the variability of vital signs, such as mean arterial pressure, heart rate, and cardiac output, enable earlier diagnosis of traumatic hemorrhagic shock, thereby increasing the likelihood of timely treatment and a more favorable outcome for the patient. Although several models for predicting critical bleeding do exist, none however have been deemed sufficiently accurate to dictate treatment [42].

Among the models for predicting hemorrhagic shock, a number of these, like ours, examine the relationship between blood loss and clinical signs in order to detect it at an early stage, prior to the onset of full-blown shock. In their recent systematic reviews, Pacagnella et al. [43] and Olaussen et al. [42] outline the various available models and report on their performances and validation.

More recently, Chen et al. [44] proposed a hemorrhage identification model using non-invasive vital signs. Vital signs remain the most important predictors of hemorrhagic shock before admittance to hospitals, such as on the battlefield, at the scene of terrorist acts or motor vehicle accidents.

Furthermore, HR, systolic blood pressure (SBP), and MAP allow one to calculate the so-called shock index (SI), i.e., the HR/SBP ratio, and the modified SI (MSI), which is the HR/MAP ratio. These are by far the most sensitive markers of hypoperfusion and strong predictors of outcome.

With elevations of the SI above 0.9, the risk of recurring to MT in trauma patients rises substantially [45]. As soon as SI surpasses 0.9, the risk doubles; it increases fivefold for SI > 1.1 and is 7 times higher for SI > 1.3 [45]. SI is an easily measured hemodynamic indicator of instability [46–48] used to triage hypovolemic shock upon arrival at the emergency department (ED), in terms of transfusion requirements and hemostatic resuscitation [49].

However, in a recent large prospective study of 9,860 adult trauma patients, MSI (>1.3 or <0.7) was a better predictor of mortality than conventional SI, implying that mean, rather than systolic, BP is what ensures tissue perfusion [50].

Given the fact that our model also simulates the performance of HR and MAP, it too proves itself capable of predicting hemorrhagic shock, representing a useful option in the assessment of MT requirements, based on MSI, for traumatized patients.

### 4.1. Limitations of the Current Work

The model, in its current configuration, is inadequate for simulating baroreflex mechanisms. Further study is necessary in order to expand the algorithm to be able to account for effects of variations in stress scenarios on baroreflex responses.

Another, albeit intentional, limitation of the current work is the relative simplicity of the representation of the vascular circuit.

The introduction of a more sophisticated mathematical structure, perhaps including the pulmonary circulation, while offering a better representation, would however overburden the computational calculation times, at the expense of (non-negligible) delays in output.

An important limitation consists in not having used conscious swine: the anesthetics used during the implementation of porcine hemorrhage models for the hemorrhagic shock undoubtedly introduce some confounding effects. In particular, isoflurane (used by us) is an anesthetic that attenuates transcapillary refill after both normotensive and hypotensive hemorrhage [51].

Intuitively, the rationale for administering anesthetic agents is to spare lab animals unnecessary pain when surgical procedures are required for a study, as is often the case. Such uses are not only warranted but also mandated, according to established ethical protocols recognized internationally for the use of animal models in any experimental setting [20].

As regards future research, the criteria for selecting anesthetic agents will favor those having minimal physiologic effects on normal compensatory responses to blood loss as we pursue further validation of the model. As of yet, the model has only been tested (with encouraging results) on a small cohort of 4 laboratory swine, but it is clear that subsequent validation will imply larger cohorts in order to produce more quantitatively representative results, as well as increasingly closer to the conscious threshold.

## 5. Conclusion

In the current study, we introduce an upgrade of Zenker's model, dubbed the ZenCur model. While maintaining the mathematical structure of the original model, ours also integrates the homeostatic mechanism of transcapillary refill, a fundamental compensatory mechanism during bleeding.

The addition of this “component” enhances the performance of the system, while maintaining the relative simplicity of the model. Comparisons between our model and the experimental data from 4 laboratory animals (swine) subjected to controlled hemorrhage allowed us to establish that the ZenCur model was able to acceptably simulate the curves of the three hemodynamic variables of interest, i.e., mean arterial pressure, heart rate, and cardiac output, in the context of severe bleeding.

The purpose of our study was to develop a simple and effective tool that is able to predict hemorrhagic shock in traumatized patients in a timely manner: by calculating the MSI index (which is the ratio HR/MAP), we have a highly valuable marker to predict mortality, which significantly outperforms HR, SBP, DBP, or SI alone [52].

In clinical practice, in addition to predicting survival, our system also allows us to determine the best timing of MT for the individual patient.

Upon further validation in larger cohorts, the ZenCur model has the potential of a novel, user-friendly decision-making tool. The foreseeable applications might include various scenarios such as emergency care for motor vehicle accidents, for natural disasters, or on the battlefield, once integrated into dedicated (and portable) devices that facilitate triage in the field.

## Figures and Tables

**Table 1:** Model variables.

Variable	Units	Meaning
*P*_A_	mmHg	Arterial pressure
*S*	#	Sympathetic nervous activity
*S*_*E*_	#	Stimulating output from baroreflex central processing
*ν*_HR_	/min	Heart rate in beats per minute
*R*_TPR_	mmHg · min · L^−1^	Total peripheral/systemic vascular hydraulic resistance
*C*_PRSW_	mmHg	Preload recruitable stroke work
*V*_Vun_	L	Venous unstressed volume at which the pressure induced by wall tension is 0 mmHg
*P*_CVP_	mmHg	Central venous pressure
*I*_C_	L/min	Arterio-venous capillary blood flow
*t*_Diast_	min	Duration of diastole
*I*_CO_	L/min	Veno-arterial flow (cardiac output)
*V*_A_	L	Arterial volume
${\tilde{V}}_{\text{ES}}$	L	Current estimate of end-systolic ventricular volume
${\tilde{V}}_{\text{ED}}$	L	Current estimate of end-diastolic ventricular volume
*P*_ED_	mmHg	Intraventricular pressure at the end of diastole
*P*_ES_	mmHg	Intraventricular pressure at the end of systole
*V*_ES_	L	End-systolic ventricular volume
*V*_ED_	L	End-diastolic ventricular volume
*I*_bleed_	L/min	External blood withdrawal from the venous compartment
*I*_refill_	L/min	Transcapillary refilling rate
*V*_V_	L	Venous volume

**Table 2:** Model parameters.

Parameter	Units	Meaning
*t*_0_	min	Starting time for numerical integration
*t*_end_	min	Final time for numerical integration
*t*_delta_	min	Time integration step
*t*_bleedstart_	min	Starting time of hemorrhage
*t*_bleedend_	min	End time of hemorrhage
*k*_VED0_	L	Ventricular volume end-diastolic at zero filling pressure
*P*_0LV_	mmHg	Passive empirical ventricular pressure
*k*_ELV_	/L	Constant characterizing the passive empirical pressure/volume relationship
*R*_valve_	mmHg · min · L^−1^	Hydraulic resistance opposing ventricular filling
*C*_A_	L · mmHg^−1^	Compliance of arterial compartment
*C*_V_	L · mmHg^−1^	Compliance of venous compartment
*V*_Aun_	L	Arterial unstressed volume at which the pressure induced by wall tension is 0 mmHg
*ν*_cut_	/min	First-order low-pass cut-off frequency (determining the time constant of the baroreflex response)
*S*_sat_	mmHg	Deviation from set point in mmHg where SNA reaches 99% saturation either way (determining the shape and maximal slope of the logistic baroreflex nonlinearity)
*P*_Aset_	mmHg	Set point for baroreflex regulation of mean arterial pressure
*V*_total_	L	Total blood volume
*F*_withdrawal_	L	External blood withdrawal from the venous compartment
*ν*_HR_min	/min	Heart rate minimum value
*ν*_HR_max	/min	Heart rate maximum value
*C*_PRSW_min	mmHg	Preload recruitable stroke work minimum value
*C*_PRSW_max	mmHg	Preload recruitable stroke work maximum value
*R*_TPR_min	mmHg · min · L^−1^	Total peripheral/systemic vascular hydraulic resistance minimum value
*R*_TPR_max	mmHg · min · L^−1^	Total peripheral/systemic vascular hydraulic resistance maximum value
*V*_Vun_Min	L	Venous unstressed volume at which the pressure induced by wall tension is 0 mmHg minimum value
*V*_Vun_Max	L	Venous unstressed volume at which the pressure induced by wall tension is 0 mmHg maximum value
*S*_F_	#	Flag: -1 for closed loop; open reflex otherwise
*k*_Vcap_	L/min/mmHg	Rate of transcapillary refill per mmHg of difference between venous and interstitial pressure
*V*_Vun0_min	L	Venous unstressed volume at which the pressure induced by wall tension is 0 mmHg minimum initial value
*V*_Vun0_max	L	Venous unstressed volume at which the pressure induced by wall tension is 0 mmHg maximum initial value
*P*_A0_	mmHg	Arterial pressure initial value
*S*_0_	#	Sympathetic nervous activity initial value
*S*_*E*0_	#	Stimulating output from baroreflex central processing initial value
*V*_A0_	L	Arterial volume at Tzero
*V*_V0_	L	Venous volume at Tzero
*V*_Vun0_	L	Venous unstressed volume at which the pressure induced by wall tension is 0 mmHg initial value
*P*_CVP0_	mmHg	Pressure in the central vein initial value
*ν*_HR0_max	/min	Maximum initial value for heart rate
*t*_Syst_	min	Duration of systole (ventricular contraction period of the cardiac cycle)
*ν*_HR0_	/min	Initial value for heart rate
*t*_Diast0_	min	Duration of diastole, endpoint of filling initial value
*C*_PRSW0_	mmHg	Preload recruitable stroke work initial value
*R*_TPR0_	mmHg · min · L^−1^	Total peripheral/systemic vascular hydraulic resistance initial value
*I*_C0_	L · min^−1^	Arterio-venous capillary blood flow initial value
*I*_CO0_	L · min^−1^	Veno-arterial flow (cardiac output) initial value
*V*_ES0_	L	End-systolic ventricular volume initial value
*V*_ED0_	L	End-diastolic ventricular volume initial value
${\tilde{V}}_{\text{ES}0}$	L	End-systolic ventricular volume tilde initial value
${\tilde{V}}_{\text{ED}0}$	L	End-diastolic ventricular volume tilde initial value
*P*_ED0_	mmHg	End-diastolic ventricular pressure initial value
*P*_ES0_	mmHg	End-systolic ventricular pressure initial
*I*_bleed0_	L · min^−1^	External blood withdrawal from the venous compartment initial value
*I*_refill0_	L/min	Transcapillary refilling rate initial

**Table 3:** ZenCur model parameter configurations, Figures 2–4.

Parameter	Units	Subject 1	Subject 2	Subject 3	Subject 4
^⋄^*t*_0_	min	0	0	0	0
^⋄^*t*_end_	min	500	500	500	500
^⋄^*t*_delta_	min	0.01	0.01	0.01	0.01
°*t*_bleedstart_	min	40	40	25	33.3
°*t*_bleedend_	min	73	73	53	50
^†^*k*_VED0_	L	0.00714414	0.00714414	0.00714414	0.00714414
^†^*P*_0LV_	mmHg	2.03247	2.03247	2.03247	2.03247
^†^*k*_ELV_	/L	65.6531	65.6531	65.6531	65.6531
^∗^*R*_valve_	mmHg · min · L^−1^	0.0416667	0.0416667	0.0416667	0.0416667
^∗^*C*_A_	L · mmHg^−1^	0.0031	0.0031	0.0031	0.0011
^∗^*C*_V_	L · mmHg^−1^	0.05083	0.101	0.05083	0.0973
^∗^*V*_Aun_	L	0.17072	0.21	0.17072	0.21
^∗^*ν*_cut_	/min	0.00252	0.000192	0.0048	0.000282
^∗^*S*_sat_	mmHg	25	25	25	25
^∗^*P*_Aset_	mmHg	68.3393	80.4533	69.4119	73
^‡^*V*_total_	L	2.85	4.29	2.85	4.29
°*F*_withdraw*al*_	L	0.92	1.56	0.96	1.73
^∗^*ν*_HR_min	/min	0	15	0	0
^∗^*ν*_HR_max	/min	166	166.2	170	166.2
^∗^*C*_PRSW_min	mmHg	14.509	60.509	14.509	30.509
^∗^*C*_PRSW_max	mmHg	130.1	85.1	130.1	65.1
^∗^*R*_TPR_min	mmHg · min · L^−1^	14.8333	7.8333	14.8333	16.8333
^∗^*R*_TPR_max	mmHg · min · L^−1^	19.8333	30.8333	19.8333	18.8
^∗^*V*_Vun_Min	L	1.47	1.9	1.47	1.66
^∗^*V*_Vun_Max	L	1.625	2	1.625	2.15
^⋄^*S*_*F*_	#	-1	-1	-1	-1
^∗^*k*_Vcap_	L/min/mmHg	2.1*e* − 05	0	1.7*e* − 05	1*e* − 07
^★^*V*_Vun0_min	L	1.47	1.9	1.47	1.66
^★^*V*_Vun0_max	L	1.625	2	1.625	2.15
^★^*P*_A0_	mmHg	68.3393	80.4533	69.4119	73
^★^*S*_0_	#	0.5	0.5	0.5	0.5
^★^*S*_*E*0_	#	0.5	0.5	0.5	0.5
^★^*V*_A0_	L	0.382572	0.459405	0.385897	0.2903
^★^*V*_V0_	L	2.46743	3.83059	2.4641	3.9997
^★^*V*_Vun0_	L	1.5475	1.95	1.5475	1.905
^★^*P*_CVP0_	mmHg	18.0981	18.6198	18.0327	21.5283
^★^*ν*_HR0_max	/min	166	166.2	170	166.2
^★^*t*_Syst_	min	0.00481928	0.00481348	0.00470588	0.00481348
^★^*ν*_HR0_	/min	83	90.6	85	83.1
^★^*t*_Diast0_	min	0.00722892	0.00622405	0.00705882	0.00722022
^★^*C*_PRSW0_	mmHg	72.3045	72.8045	72.3045	47.8045
^★^*R*_TPR0_	mmHg · min · L^−1^	17.3333	19.3333	17.3333	17.8166
^★^*I*_C0_	L · min^−1^	2.89853	3.19829	2.96419	2.88897
^★^*I*_CO0_	L · min^−1^	2.89853	3.19829	2.96419	2.88897
^★^*V*_ES0_	L	0.00714414	0.00714414	0.00714414	0.00714414
^★^*V*_ED0_	L	0.0420662	0.0424454	0.042017	0.0419091
${\;}^{★}{\tilde{V}}_{\text{ES}0}$	L	0.00714414	0.00714414	0.00714414	0.00714414
${\;}^{★}{\tilde{V}}_{\text{ED}0}$	L	0.0420662	0.0424454	0.042017	0.0419091
^★^*P*_ED0_	mmHg	18.0932	18.6005	18.0282	17.8867
^★^*P*_ES0_	mmHg	0	0	0	0
^★^*I*_bleed0_	L · min^−1^	0	0	0	0
^★^*I*_refill0_	L/min	0	0	0	0
*J*	#	2.0389	1.7329	4.3471	2.6835
*J*_nobase_	#	1.7425	1.5258	3.0562	2.1712

**Table 4:** Ranked (most-to-least) sensitivities for the arterial pressure *P*_A_, heart rate *ν*_HR_, and cardiac output *I*_CO_ with respect to all free parameters.

Rank	Parameter	*I*_CO_	*P*_A_	*ν*_HR_	Sensitivity
1	*V*_Vun_Min	2.291	1.334	0.031	3.656
2	*V*_Vun_Max	0.254	0.092	0.016	0.362
3	*R*_TPR_max	0.174	0.132	0.015	0.321
4	*P*_Aset_	0.214	0.052	0.042	0.308
5	*ν*_HR_max	0.167	0.033	0.066	0.266
6	*C*_V_	0.093	0.037	0.018	0.148
7	*R*_TPR_min	0.096	0.013	0.010	0.119
8	*V*_Aun_	0.076	0.027	0.013	0.116
9	*C*_A_	0.042	0.030	0.011	0.083
10	*ν*_HR_min	0.004	0.011	0.061	0.076
11	*ν*_cut_	0.024	0.010	0.005	0.039
12	*S*_sat_	0.008	0.007	0.017	0.032
13	*k*_Vcap_	0.007	0.002	0.002	0.011
14	*C*_PRSW_max	0.003	0.005	0.002	0.010
15	*C*_PRSW_min	0.002	0.003	0.002	0.007
16	*R*_valve_	0	0.002	0	0.002

**Table 5:** Baseline animals' characteristics (means ± SDs).

Animals' characteristics	Units	Evaluated pigs
Weight	kg	46.5 ± 5.9
Cardiac output	mL/kg	98.7 ± 26.4
Heart rate	beats/min	86.8 ± 3.5
Mean arterial pressure	mmHg	66.8 ± 11.1
Systolic arterial pressure	mmHg	79.8 ± 9.9
Diastolic arterial pressure	mmHg	54.3 ± 9.4
Pulse pressure	mmHg	25.5 ± 2.5
Central venous pressure	mmHg	7.5 ± 6.4
Mean pulmonary arterial pressure	mmHg	13.5 ± 5.8

## Data Availability

All relevant data are within the paper.

## Appendix

### A. Cardiovascular Model Equations and Initial Conditions

Below, we provide the complete set of equations for Zenker's cardiovascular model [12]:

#### A.1 Circulatory Model

The stroke volume is

(25) $$\begin{array}{c} V_{\text{S}}=V_{\text{ED}}-V_{\text{ES}}. \end{array}$$

${\tilde{V}}_{\text{ES}}$ is the current estimate of end-systolic ventricular volume.

(26) $$\begin{array}{c} {\tilde{V}}_{\text{ES}}\left(V_{\text{ED}}\right)=\left\{\begin{array}{c} \max\left(k_{\text{VED}0},{\hat{V}}_{\text{ES}}\left(V_{\text{ED}}\right)\right),\;\text{if }P_{\text{A}}>P_{\text{LV}}\left(V_{\text{ED}}\right)=P_{\text{ED},}\\ k_{\text{VED}0},\;\text{otherwise}, \end{array}\right. \end{array}$$

where

(27) $$\begin{array}{c} {\hat{V}}_{\text{ES}}\left(V_{\text{ED}}\right)=V_{\text{ED}}-\frac{c_{\text{PRSW}}\left(V_{\text{ED}}-k_{\text{VED}0}\right)}{P_{\text{A}}-P_{\text{ED}}}. \end{array}$$

Substituting, we obtain

(28) $$\begin{array}{c} {\tilde{V}}_{\text{ES}}\left(V_{\text{ED}}\right)=\left\{\begin{array}{cc} \max\left(k_{\text{VED}0},V_{\text{ED}}-\frac{C_{\text{PRSW}}\cdot \left(V_{\text{ED}}-k_{\text{VED}0}\right)}{P_{\text{A}}-P_{\text{ED}}}\right), & \text{if }P_{\text{A}}>P_{\text{LV}}\left(V_{\text{ED}}\right)=P_{\text{ED}},\\ k_{\text{VED}0}, & \text{otherwise}. \end{array}\right. \end{array}$$

The dependence of ventricular pressure on ventricular volume is governed by the experimentally characterized [28] exponential relationship:

(29) $$\begin{array}{c} P_{\text{LV}}\left(V_{\text{LV}}\right)=P_{0_{\text{LV}}}\left(e^{k_{{\text{E}}_{\text{LV}}}\left(V_{\text{LV}}-k_{\text{VED}0}\right)}-1\right). \end{array}$$

*P*_ED_ is the intraventricular pressure at the end of diastole and is experimentally characterized [28] by the following exponential relationship:

(30) $$\begin{array}{c} P_{\text{ED}}=P_{0LV}\cdot \left(e^{k_{\text{ELV}}\cdot \left(V_{\text{ED}}-k_{\text{VED}0}\right)}-1\right). \end{array}$$

Substituting the term *V*_ES_ for *V*_ED_ in the above equation, we obtain *P*_ES_, that is, the intraventricular pressure at the end of systole:

(31) $$\begin{array}{c} P_{\text{ES}}=P_{0LV}\cdot \left(e^{k_{\text{ELV}}\cdot \left(V_{\text{ES}}-k_{\text{VED}0}\right)}-1\right). \end{array}$$

Ventricular filling is modeled as a filling through the linear inflow resistance *R*_valve_:

(32) $$\begin{array}{c} \frac{dV_{\text{LV}}}{dt}=\frac{P_{\text{CVP}}-P_{\text{LV}}\left(V_{\text{LV}}\right)}{R_{\text{value}}}. \end{array}$$

Under the assumption of constant *P*_CVP_, the previous equation becomes of the general form:

(33) $$\begin{array}{c} \frac{dV}{dt}=k_{1}e^{k_{2}V}+k_{3}. \end{array}$$

Considering the constants [53]:

(34) $$\begin{array}{c} k_{1}=-\frac{P_{{\text{O}}_{\text{LV}}}}{R_{\text{valve}}}e^{-k_{{\text{E}}_{\text{LV}}}k_{\text{VED}0}},\\ k_{2}=k_{{\text{E}}_{\text{LV}}},\\ k_{3}=\frac{P_{\text{CVP}}+P_{0_{\text{LV}}}}{R_{\text{valve}}}, \end{array}$$

and assuming *t* = 0 as the beginning of diastole and employing end-systolic volume *V*_ES_ as the initial condition, Zenker and coauthors obtain this solution:

(35) $$\begin{array}{c} V\left(t\right)\;=-\frac{1}{k_{2}}\ln\;\left(\frac{k_{1}}{k_{3}}\left(e^{-k_{2}k_{3}t}-1\right)+e^{-k_{2}\left(V_{ES}+k_{3}t\right)}\right). \end{array}$$

At a given heart rate *ν*_HR_, and assuming a constant duration of systole *T*_Sys_ [54], the end-diastolic volume will therefore be

(36) $$\begin{array}{c} {\hat{V}}_{\text{ED}}=V\left(f_{\text{HR}}^{-1}-t_{\text{Sys}}\right), \end{array}$$

with *V*(*t*) given by the previous equation and *t*_Diast_ is given by

(37) $$\begin{array}{c} t_{\text{Diast}}=\frac{1}{\nu_{\text{HR}}}-t_{\text{Syst}.} \end{array}$$

${\tilde{V}}_{\text{ED}}$ is the current estimate of end-diastolic ventricular volume:

(38) $$\begin{array}{c} {\tilde{V}}_{\text{ED}}\left(V_{\text{ES}}\right)=\left\{\begin{array}{c} {\hat{V}}_{\text{ED}},\;\text{if }P_{\text{CVP}}>P_{\text{LV}}\left(V_{\text{ES}}\right)=P_{\text{ES}},\\ V_{\text{ES}},\;\text{otherwise}, \end{array}\right. \end{array}$$

by making the appropriate replacements:

(39) $$\begin{array}{c} {\tilde{V}}_{\text{ED}}\left(V_{\text{ES}}\right)=\left\{\begin{array}{cc} -\frac{1}{k_{\text{ELV}}}\cdot \log\left(-\frac{P_{0LV}}{P_{\text{CVP}}+P_{0\text{LV}}}\cdot e^{-k_{\text{ELV}}\cdot k_{\text{VED}0}}\cdot \left(e^{-k_{\text{ELV}}\cdot \left(\left(P_{\text{CVP}}+P_{0\text{LV}}\right)/R_{\text{valve}}\right)\cdot t_{\text{Diast}}}-1\right)+e^{-k_{\text{ELV}}\cdot \left(V_{\text{ES}}+\left(\left(P_{\text{CVP}}+P_{0\text{LV}}\right)/R_{\text{valve}}\right)\cdot t_{\text{Diast}}\right)}\right), & \text{ if }P_{\text{CVP}}>P_{\text{LV}}\left(V_{\text{ES}}\right)=P_{\text{ES}},\\ V_{\text{ES}}, & \text{otherwise}. \end{array}\right. \end{array}$$

*V*_ES_ is the end-systolic ventricular volume and is evaluated as

(40) $$\begin{array}{c} \frac{dV_{\text{ES}}}{dt}=\left({\tilde{V}}_{\text{ES}}-V_{\text{ES}}\right)\cdot \nu_{\text{HR}}. \end{array}$$

*V*_ED_ is the end-diastolic ventricular volume that is described by the following differential equation:

(41) $$\begin{array}{c} \frac{dV_{\text{ED}}}{dt}=\left({\tilde{V}}_{\text{ED}}-V_{\text{ED}}\right)\cdot \nu_{\text{HR}}. \end{array}$$

*P*_A_ is the arterial pressure:

(42) $$\begin{array}{c} P_{\text{A}}=\frac{V_{\text{A}}-V_{\text{Aun}}}{C_{\text{A}}}, \end{array}$$

where *V*_A_ is the arterial volume, *V*_Aun_ is the arterial volume unstressed, and *C*_A_ is the arterial compliance.

*P*_CVP_ is the central venous pressure evaluated as the ratio between the venous volume *V*_V_ minus the unstressed venous volume *V*_Vun_ and venous compliance *C*_V_:

(43) $$\begin{array}{c} P_{\text{CVP}}=\frac{V_{\text{V}}-V_{\text{Vun}}}{C_{\text{V}}}. \end{array}$$

*I*_C_ is the arterio-venous capillary blood flow, i.e., the total of blood volume per minute traversing the whole systemic capillary bed

(44) $$\begin{array}{c} I_{\text{C}}=\frac{P_{\text{A}}-P_{\text{CVP}}}{R_{\text{TPR}}}. \end{array}$$

*I*_CO_ is the cardiac output that represents the total flow generated by the heart per unit time:

(45) $$\begin{array}{c} I_{\text{CO}}=\left(V_{\text{ED}}-V_{\text{ES}}\right)\cdot \nu_{\text{HR}}, \end{array}$$

where *V*_ED_ is the end-diastolic volume and *V*_ES_ is the end-systolic volume.

*V*_A_ is the arterial volume and is evaluated as

(46) $$\begin{array}{c} \frac{dV_{\text{A}}}{dt}=I_{\text{CO}}-I_{\text{C}}, \end{array}$$

*V*_V_ is the venous volume and is determined by the following differential equation, which takes into account the temporal variation in arterial blood volume, rate of capillary refill, and bleeding rate:

(47) $$\begin{array}{c} \frac{dV_{\text{V}}}{dt}=-\frac{dV_{\text{A}}}{dt}+I_{\text{external}}=-\frac{dV_{\text{A}}}{dt}+I_{\text{bleed}}+I_{\text{refill}}=I_{\text{C}}-I_{\text{CO}}+I_{\text{bleed}}+I_{\text{refill}}. \end{array}$$

*I*_bleed_ is the rate of external hemorrhage from the venous compartment during a simulated or experimental bleed:

(48) $$\begin{array}{c} I_{\text{bleed}}=\left\{\begin{array}{cc} -\frac{F_{\text{withdrawal}}}{t_{\text{bleedend}}-t_{\text{bleedstart}}}, & \text{if }t\geq t_{\text{bleedstart}}\text{ and }t\leq t_{\text{bleedend}},\\ 0, & \text{otherwise}, \end{array}\right. \end{array}$$

where *F*_withdrawal_ is the volume of blood lost and *t*_bleedstart_ and *t*_bleedend_ are the time of initiation and termination of bleeding, respectively.

*I*_refill_ is the transcapillary refill, hypothesized to depend on the difference between central venous pressure and interstitial fluid pressure. The latter is assumed to be constant and equal to the steady state of *P*_CVP_ value (*P*_CVP0_).

(49) $$\begin{array}{c} I_{\text{refill}}=k_{\text{Vcap}}\cdot \left(P_{\text{CVP}0}-P_{\text{CVP}}\right), \end{array}$$

where *k*_Vcap_ is the proportionality constant between the rate of transcapillary and the venous-to-interstitial pressure difference.

#### A.2 Baroreflex Model

*S*_*E*_ is the stimulating output of the feedback loop determined by

(50) $$\begin{array}{c} \frac{dS_{E}}{dt}=\left\{\begin{array}{cc} 2\cdot \pi \cdot \nu_{\text{cut}}\cdot \left(S-S_{E}\right), & \text{if }S_{\text{F}}<0,\\ 0, & \text{otherwise}. \end{array}\right. \end{array}$$

The parameter *ν*_cut_ represents the first-order low-pass cut-off frequency (determining the time constant of the baroreflex response), and *S*_F_ determines whether it is an open- or closed-loop baroreflex.

*S* is the sympathetic nervous activity:

(51) $$\begin{array}{c} S=1-\frac{1}{1+e^{-\left(2\cdot \log\left(10\right)/S_{\text{sat}}\right)\left(P_{\text{A}}-P_{\text{Aset}}\right)}}, \end{array}$$

where *P*_Aset_ is the set point of baroreflex regulation regarding mean arterial pressure (in correspondence of the midpoint of the sigmoid curve, i.e., the nonlinearity of the baroreflex mechanism) and *S*_sat_ plots the upper limit of the range in which the nonlinearity of the baroreflex mechanism can operate.

The stimulating output *S*_*E*_(*t*) of the feedback loop acts on heart rate *ν*_HR_, total peripheral resistance *R*_TPR_, myocardial contractility *C*_PRSW_, and unstressed venous volume *V*_Vun_:

(52) $$\begin{array}{c} \nu_{\text{HR}}=S_{E}\cdot \left(\nu_{\text{HR}}\max-\nu_{\text{HR}}\min\right)+\nu_{\text{HR}}\min, \end{array}$$

where *ν*_HR_max and *ν*_HR_min are the maximum and minimum heart rates, respectively.

(53) $$\begin{array}{c} R_{\text{TPR}}=S_{E}\cdot \left(R_{\text{TPR}}\max-R_{\text{TPR}}\min\right)+R_{\text{TPR}}\min, \end{array}$$

where *R*_TPR_max and *R*_TPR_min are the maximum and minimum values for the peripheral systemic vascular hydraulic resistance, respectively.

(54) $$\begin{array}{c} C_{\text{PRSW}}=S_{E}\cdot \left(C_{\text{PRSW}}\max-C_{\text{PRSW}}\min\right)+C_{\text{PRSW}}\min, \end{array}$$

where *C*_PRSW_max and *C*_PRSW_min are the maximum and minimum values for the preload recruitable stroke work, respectively.

(55) $$\begin{array}{c} V_{\text{Vun}}=\left(1-S_{E}\right)\cdot \left(V_{\text{Vun}}\max-V_{\text{Vun}}\min\right)+V_{\text{Vun}}\min, \end{array}$$

where *V*_Vun_min and *V*_Vun_max are the maximum and minimum values for the venous unstressed volume, respectively.

The set of five ODE describing the cardiovascular system:

(56) $$\begin{array}{c} \begin{array}{c} \frac{dV_{\text{ES}}}{dt}=\left({\tilde{V}}_{\text{ES}}\left(V_{\text{ED}},V_{\text{A}},S_{E}\right)-V_{\text{ES}}\right)\nu_{\text{HR}}\left(S_{E}\right),\\ \frac{dV_{\text{ED}}}{dt}=\left({\tilde{V}}_{\text{ED}}\left(V_{\text{ES}},V_{\text{V}}\right)-V_{\text{ED}}\nu_{\text{HR}}\left(S_{E}\right)\right.,\\ \frac{dV_{\text{A}}}{dt}=\frac{P_{\text{A}}\left(V_{\text{A}}\right)-P_{\text{V}}\left(V_{\text{V}},S_{E}\right)}{R_{\text{TPR}}\left(S_{E}\right)}-\left(V_{\text{ED}}-V_{\text{ES}}\right)\nu_{\text{HR}}\left(S_{E}\right),\\ \frac{dV_{\text{V}}}{dt}=-\frac{dV_{\text{A}}}{dt}+I_{\text{external}}\left(t\right),\\ \frac{dS_{E}}{dt}=2\cdot \pi \cdot \nu_{\text{cut}}\cdot \left(S-S_{E}\right). \end{array} \end{array}$$

#### A.3 Initial Conditions

Parameter values obtained taking into account the steady-state conditions:

(57) $$\begin{array}{c} V_{\text{Vun}0}\min=V_{\text{Vun}}\mathrm{Min},\\ V_{\text{Vun}0}\max=V_{\text{Vun}}\mathrm{Max},\\ P_{\text{A}0}=P_{\text{Aset}}. \end{array}$$

The sympathetic nervous activity is computed directly from the definition at *t*_0_, when *P*_A_ = *P*_A0_ and *P*_A0_ = *P*_Aset_:

(58) $$\begin{array}{c} S_{0}=1-\frac{1}{1+e^{-\left(2\cdot \log\left(10\right)/S_{\text{sat}}\right)\left(P_{\text{A}0}-P_{\text{Aset}}\right)}},\\ S_{E0}=S_{0},\\ V_{\text{A}0}=P_{\text{A}0}\cdot C_{\text{A}}+V_{\text{Aun}},\\ V_{\text{V}0}=V_{\text{total}}-V_{\text{A}0},\\ V_{\text{Vun}0}=\left(1-S_{E0}\right)\cdot \left(V_{\text{Vun}}\mathrm{Max}-V_{\text{Vun}}\mathrm{Min}\right)+V_{\text{Vun}}\mathrm{Min},\\ P_{\text{CVP}0}=\frac{V_{\text{V}0}-V_{\text{Vun}0}}{C_{\text{V}}}. \end{array}$$

The maximum initial value for the heart rate is equal to the maximum value of the heart rate:

(59) $$\begin{array}{c} \nu_{\text{HR}0}\max=\nu_{\text{HR}}\max,\\ t_{\text{Syst}}=\frac{1}{\nu_{\text{HR}0}\max}\cdot 0.8,\\ \nu_{\text{HR}0}=S_{E0}\cdot \left(\nu_{\text{HR}}\max-\nu_{\text{HR}}\min\right)+\nu_{\text{HR}}\min,\\ t_{\text{Diast}0}=\frac{1}{\nu_{\text{HR}0}\max}-t_{\text{Syst}},\\ C_{\text{PRSW}0}=S_{E0}\cdot \left(C_{\text{PRSW}}\max-C_{\text{PRSW}}\min\right)+C_{\text{PRSW}}\min,\\ R_{\text{TPR}0}=S_{E0}\cdot \left(R_{\text{TPR}}\max-R_{\text{TPR}}\min\right)+R_{\text{TPR}}\min,\\ I_{\text{C}0}=\frac{P_{\text{A}0}-P_{\text{CVP}0}}{R_{\text{TPR}0}},\\ I_{\text{CO}0}=I_{\text{C}0},\\ V_{\text{ES}0}=k_{\text{VED}0},\\ V_{\text{ED}0}=V_{\text{ES}0}+\frac{I_{\text{C}0}}{\nu_{\text{HR}0}},\\ {\tilde{V}}_{\text{ES}0}=V_{\text{ES}0},\\ {\tilde{V}}_{\text{ED}0}=V_{\text{ED}0},\\ P_{\text{ED}0}=P_{0LV}\cdot \left(e^{k_{\text{ELV}}\cdot \left(V_{\text{ED}0}-k_{\text{VED}0}\right)}-1\right),\\ P_{\text{ES}0}=P_{0LV}\cdot \left(e^{k_{\text{ELV}}\cdot \left(V_{\text{ES}0}-k_{\text{VED}0}\right)}-1\right),\\ I_{\text{bleed}0}=0,\\ I_{\text{refill}0}=0. \end{array}$$
